# Changes in the lipidome of water buffalo milk during intramammary infection by non-aureus Staphylococci

**DOI:** 10.1038/s41598-022-13400-0

**Published:** 2022-06-11

**Authors:** Fabrizio Ceciliani, Matteo Audano, Maria Filippa Addis, Nico Mitro, Cristina Lecchi, Morteza H. Ghaffari, Mariangela Albertini, Esterina De Carlo, Domenico Vecchio, Gabriele Di Vuolo, Giovanna Cappelli, Francesco Tangorra, Renata Piccinini, Valerio Bronzo, Donatella Caruso

**Affiliations:** 1 Department of Veterinary Medicine, Università degli Studi di Milano, Via dell’Università 6, 26900 Lodi, Italy; 2 Department of Pharmacological and Biomolecular Sciences, Università degli Studi di Milano, Via Balzaretti, 9/11/13, 20133 Milan, Italy; 3 Institute for Animal Science, Physiology Unit, University of Bonn, Katzenburgweg 7-9, 53115 Bonn, Germany; 4 Italian National Reference Centre for Hygiene and Technologies of Water Buffalo Farming and Productions (CReNBuf), Istituto Zooprofilattico Sperimentale del Mezzogiorno, 80055 Portici, Italy

**Keywords:** Immunology, Microbiology, Systems biology

## Abstract

This study aimed to determine the lipidome of water buffalo milk with intramammary infection (IMI) by non-aureus staphylococci (NAS), also defined as coagulase-negative staphylococci, using an untargeted lipidomic approach. Non-aureus Staphylococci are the most frequently isolated pathogens from dairy water buffalo milk during mastitis. A total of 17 milk samples from quarters affected by NAS-IMI were collected, and the lipidome was determined by liquid chromatography-quadrupole time-of-flight mass spectrometry. The results were compared with the lipidome determined on samples collected from 16 healthy quarters. The study identified 1934 different lipids, which were classified into 15 classes. The abundance of 72 lipids changed in NAS-IMI milk compared to healthy quarters. Significant changes occurred primarily in the class of free fatty acids. The results of this study provided first-time insight into the lipidome of dairy water buffalo milk. Moreover, the present findings provide evidence that NAS-IMI induces changes in water buffalo milk's lipidome.

## Introduction

Water buffaloes provide the most significant proportion of non-cattle milk globally (15.14%), and in some countries they are the most important source of milk for human consumption^1^. Dairy water buffaloes can be affected by mastitis, which is only slightly less common than in cows^2,3^, although information about the pathogens involved is limited. Among the organisms related to intramammary infection, non-aureus staphylococcus (NAS) are the most commonly isolated group of pathogens causing intramammary infections in cattle and buffalo, causing 78% of intramammary infections^4–8^. Exploring the impact of NAS in water buffalo milk is crucial, given the background that several studies identified NAS in water buffalo milk as a source of antibiotic resistance^8–12^.

Despite previous studies using classical methods^3,13,14^, OMICS technologies may provide a more informative picture of mastitis in water buffalo. A limited number of studies have used OMICS technologies to study water buffalo mastitis, except for microbiomics^15–18^ and proteomics. Most studies have examined the proteome with a focus on milk quality in food adulteration and nutritional value^19–21^. Two recent proteomic studies found changes in the milk proteome in water buffalo with subclinical mastitis^22,23^. Transcriptome analysis of milk revealed differences in the transcriptome of somatic cells of Murrah buffaloes and Sahiwal cattle^24^. Studies on lipidomics are lacking. The few data available focus on food quality^25^ and reproduction^26^. To the best of the author's knowledge, no studies have been conducted to characterize the lipidome of water buffalo milk using the OMIC approach. Lipids molecules play an essential role in all biological processes, including energy storage, cell membranes formation, and signal transmission. Lipids component of milk includes mediators of inflammation, such as oxylipids, potent regulators of bovine mammary gland inflammation^27,28^.

This study aimed to determine the lipidome of water buffalo milk with NAS intramammary infection (NAS-IMI) using an untargeted liquid chromatography-quadrupole time-of-flight mass spectrometry (LC -QTOF- MS) approach.

## Results

### Analytical workflow, animal classification, and disease diagnosis

**Figure 1:**
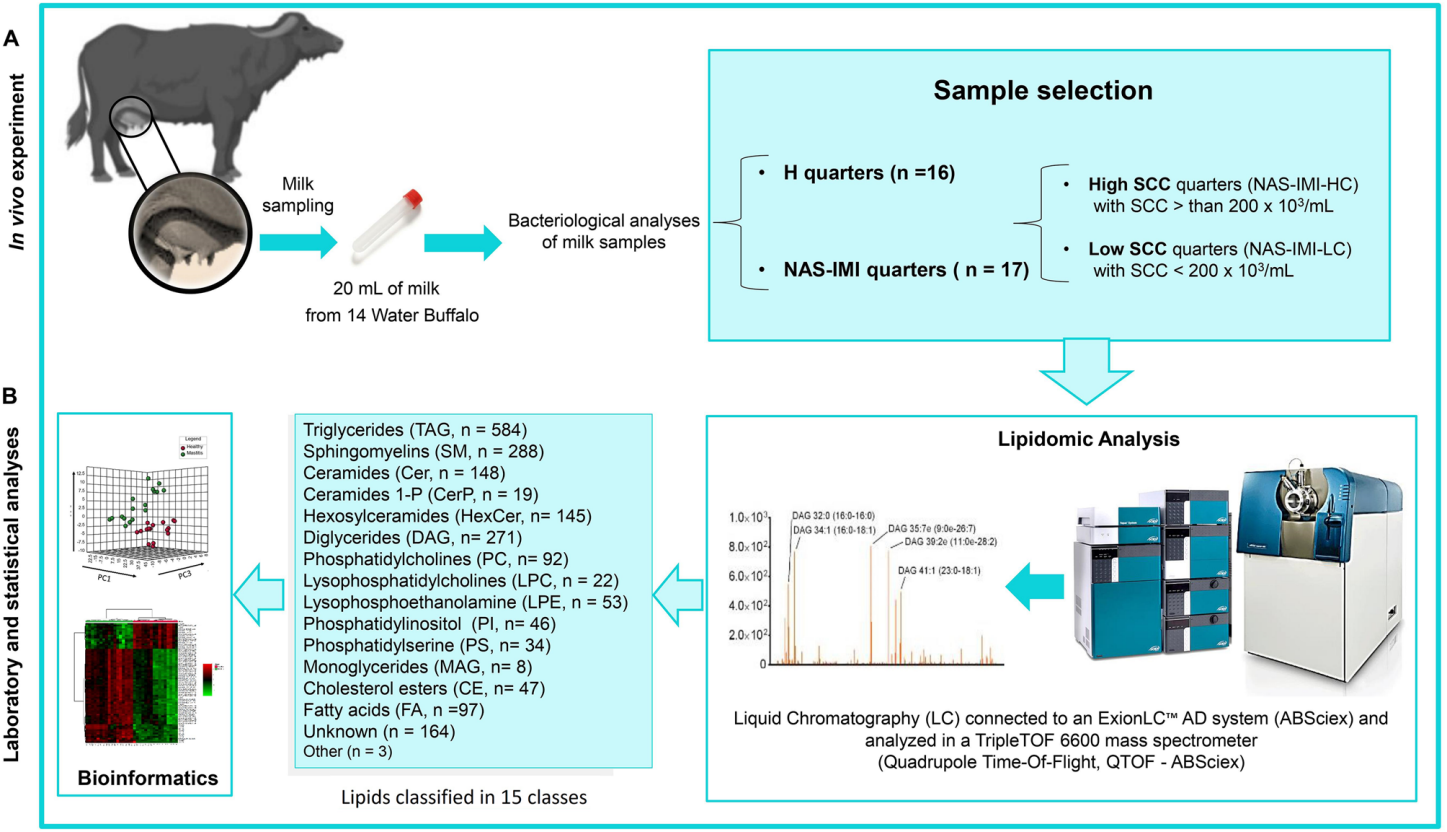
Schematic of the experimental design. Milk was collected from healthy and NAS-IMI quarters of water buffaloes and processed for lipidomic analysis through a liquid chromatography quadrupole time-of-flight (LC-Q-TOF) mass spectrometer. The figure was created using the web-based tool BioRender (https://app.biorender.com) to generate Fig. 1.

Figure 1 presents the analytical workflow applied to investigate H and NAS-IMI quarter milk samples, the characteristics of which are described in Supplementary Table S1. Given that the SCC of three milk samples was higher than 100 × 10^3^/mL, the healthy quarters were further classified into low SCC (< 100 × 10^3^/mL—HLC – 14 samples) and high SCC (> than 100 × 10^3^/mL—3 samples: HSSC7, HSSC8, and HSSC9).

### The lipidome of healthy water buffalo milk

A total of 1934 lipid species were identified in the untargeted lipidome of water buffalo milk. According to Table 1, lipids were classified into 15 classes, along with pantothenic acid. Detailed information on identified lipid species, including retention time, ionisation mode, adduct ions, and the mass difference between experimental and theoretical mass, is presented in Table S2. It must be remarked that automated annotation of metabolites and lipids is a significant issue and identifications via these tools remains tentative.

**Table 1:** The lipidome of water buffalo milk.

Lipid class	Acronym	Lipid species in water buffalo milk
Triacylglycerols	TAG	584
Diacylglycerols	DAG	271
Monoacylglycerols	MAG	8
Phosphatidylserines	PS	34
Sphingomyelins	SM	288
Phosphatidylinositols	PI	46
Phosphatidylcholines	PC	92
Lysophosphatidylethanolamines	LPE	53
Phatidylethanolamines	PE	80
Lysophosphatidylcholines	LPC	22
Ceramides	Cer	148
Ceramides 1 phosphate	CerP	19
Cholesterol esters	CE	47
Hexoceramide	HexCer	145
Free fatty acids	FA	97

**Figure 2:**
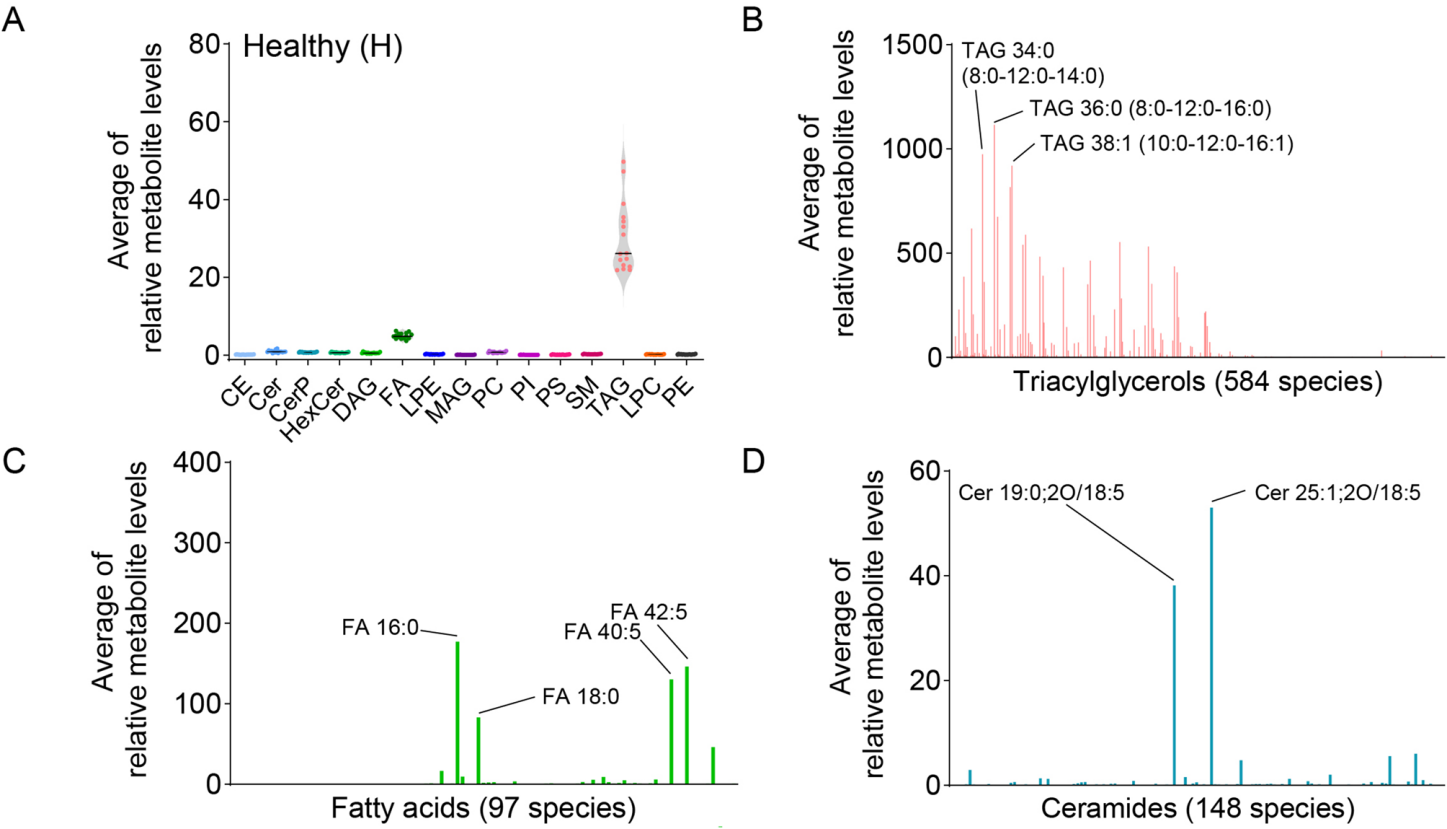
TAG, FA and Cer are the most abundant lipid classes in healthy buffalo milk samples. (**A**) Relative abundance of the main lipid classes in healthy buffalo milk samples. Histograms of the most abundant lipid classes, namely (**B**) TAG, (**C**) FA, and (**D**) Cer.

Lipidomic analysis of the healthy milk showed that the abundance of TAG, FA and Cer was greater than that of the other major lipid classes, despite only FA and TAG being significantly higher compared to other classes (Fig. 2A and Supplementary Table S3). The most abundant lipids on TAG were TAG 36:0 (TAG 8:0–12:0–16:0), 38:1 (TAG 10:0–12:0–16:1), 34:0 (TAG 8:0–12:0–14:0) (Fig. 2A, B). Of the 97 species of FA, FA 16:0, FA 42:5, FA 40:5, and FA 18:0 were the most abundant (Fig. 2C). The third class that was found to be more abundant is the Cer. The two most representative lipids are Cer 25:1;2O/18:5 and Cer 19:0;2O/18:5 (Fig. 2D). The full list of identified lipids can be found in Supplementary Table S4.

This study included 16 quarters of milk samples negative on microbiological analysis and had an SCC < 100 × 10^3^, except for three quarters, HSSC7, HSSC8, HSSC13, negative on bacteriological culture but with the SCC > 100 × 10^3^. Therefore, we determined the differences between H quarters (SCC < 100,000) and HSSC quarters (SCC > 100,000) by PCA analysis. As shown in Supplementary Fig. S1, PCA showed no separation between quarter milk samples by SCC. Therefore, all 16 healthy samples were included in further analysis.

### The lipidomic profile of NAS-IMI milk quarters

**Figure 3:**
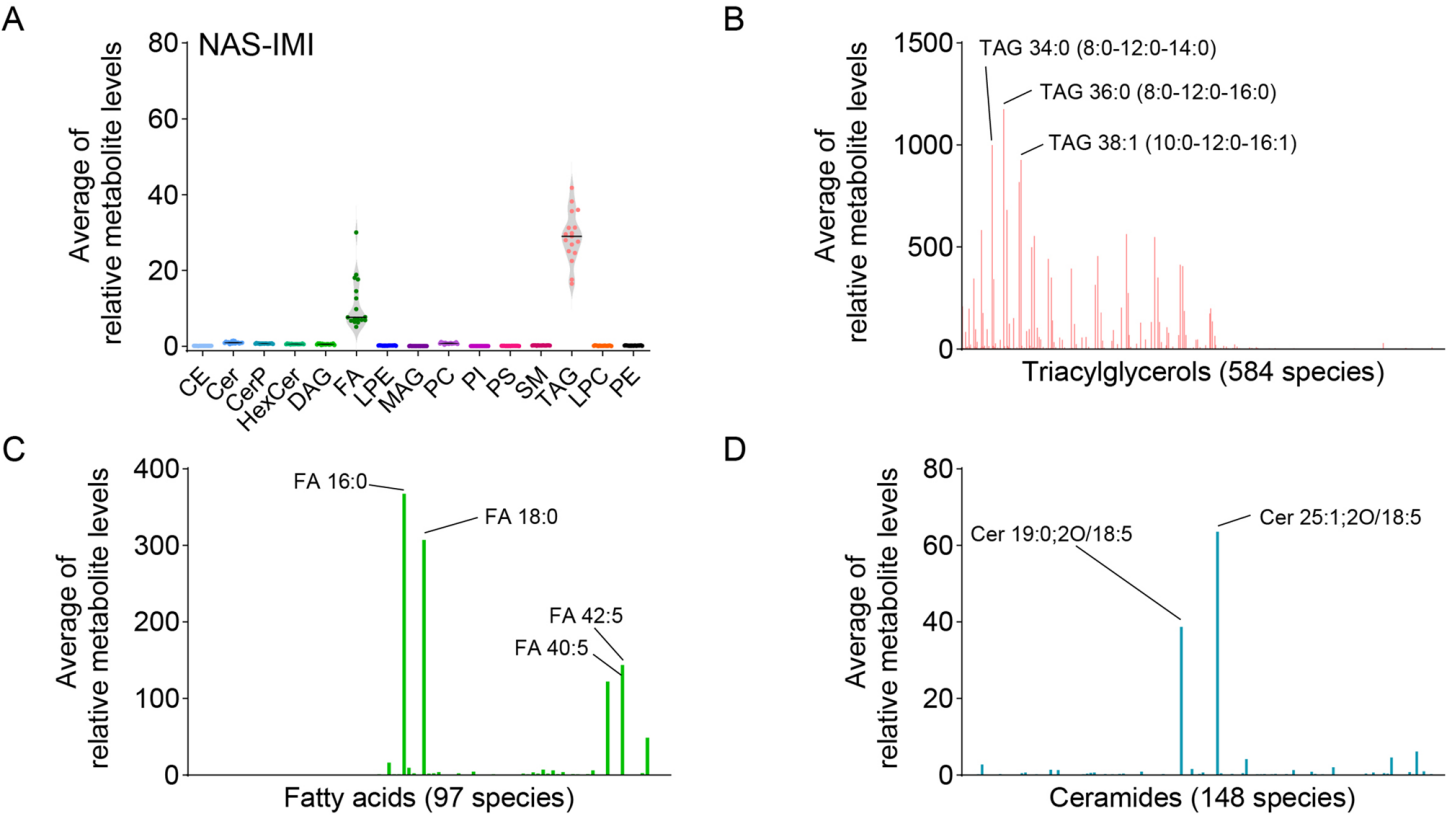
TAG, FA and Cer are the most abundant lipid classes in NAS-IMI affected milk quarter. (**A**) Relative abundance of the main lipid classes in NAS-IMI buffalo milk samples. Histograms of the most abundant lipid classes, namely (**B**) TAG, (**C**) FA, and (**D**) Cer.

The second part of the study focused on the lipidome of the milk samples from the quarters affected by NAS-IMI. The results are shown in Fig. 3.

Like the milk from healthy buffaloes, the milk from NAS-IMI affected milk contained a high TAG, FA, and Cer (Fig. 3A). Also, in NAS-IMI affected milk, TAG and FA were significantly more abundant compared to other classes, while the most abundant species were TAG 36:0 (TAG 8:0–12:0–16:0), 38:1 (TAG 10:0–12:0–16:1), 34:0 (TAG 8:0–12:0–14:0) (Fig. 3B, and Supplementary Tables S3 and S4). The significant difference is in the increase in FA, the primary species being the same as in H milk, but the relative lipid level was increased (Fig. 3C). The major FA, which changed in NAS-IMI, were FA 40:7, 20:3 and 30:0 (Table 2). For what concerns the Cer, the primary species are also Cer 25:1;2O/18:5 and Cer 19:0;2O/18:5. Significant changes are in the different relative abundance (Fig. 3D and Supplementary Table S5).

**Table 2:** Differentially abundant lipids in water buffalo milk of NAS-IMI as compared to H quarters.

Lipid	log2 FC	log10 padj	Lipid	log2 FC	log10 padj
FA 20:3	4.9668	1.4032	TAG 8:0_8:0_19:0	1.1117	6.291
FA 40:7	3.7592	6.5037	TAG 33:0|TAG 11:0_11:0_11:0	1.1096	7.3392
FA 30:0	3.5548	7.1583	TAG 57:2|TAG 19:0_19:1_19:1	1.1093	5.4509
FA 42:0	2.9227	2.3404	TAG 43:0|TAG 13:0_13:0_17:0	1.1058	6.5491
FA 17:0	2.6493	9.6397	TAG 55:1|TAG 18:0_19:0_18:1	1.1013	5.0773
FA 19:0	2.5684	10.943	TAG 31:0|TAG 9:0_9:0_13:0	1.0994	7.6256
FA 44:12	2.2375	3.1676	TAG 13:1_14:1_30:1	1.0861	4.8946
FA 25:0	2.1069	5.2216	TAG 47:1|TAG 13:0_17:0_17:1	1.0686	6.4816
FA 34:7	2.1057	5.2953	TAG 45:0|TAG 15:0_15:0_15:0	1.0683	6.5491
FA 27:0	2.0454	6.5807	FA 26:6	1.0589	3.9642
FA 28:3	1.9876	4.5099	TAG 51:2|TAG 16:0_16:0_19:2	1.0585	7.2838
FA 18:0	1.8858	8.9806	TAG 41:2|TAG 8:0_14:0_19:2	1.0564	6.5491
FA 32:7	1.877	4.9325	FA 16:0	1.0542	6.0743
FA 32:9	1.8095	9.6856	DAG 19:0_44:8	1.0497	4.9244
FA 16:4	1.7654	5.2445	TAG 51:3|TAG 14:0_18:1_19:2	1.0391	8.04
FA 21:1	1.7404	3.3105	TAG 8:0_9:0_26:3	1.0333	6.8183
FA 19:1	1.5681	3.9346	FA 20:5	1.0268	1.4065
FA 44:10	1.538	2.7352	TAG 43:2|TAG 8:0_16:0_19:2	1.0267	5.6389
FA 15:1	1.5179	2.3797	FA 40:8	1.0261	2.1979
FA 20:1	1.3636	2.4886	TAG 49:2|TAG 14:0_16:0_19:2	1.0164	6.8265
FA 18:4	1.3383	2.686	LPC 36:0	− 1.0017	2.4371
Cer 8:1;2O/42:7	1.3081	5.3885	TAG 58:6|TAG 16:0_18:1_24:5	− 1.0123	2.4397
Cer 18:1;2O/44:8	1.2634	4.5285	SM 40:4;3O	− 1.0143	4.5202
DAG 8:0_28:0	1.2603	5.4155	SM 51:2;3O	− 1.0539	3.006
FA 22:1	1.2492	2.1804	SM 15:3;2O/42:8	− 1.0606	1.3728
SM 29:0;2O/2:0	1.2442	1.3689	SM 38:2;3O	− 1.065	1.9667
Cer 26:0;2O/18:5	1.2314	6.6968	LPE 44:3	− 1.0689	4.2189
TAG 12:0_12:0_17:1	1.2311	5.3573	TAG 44:6|TAG 8:0_14:0_22:6	− 1.0896	3.9095
Cer 12:2;2O/22:1	1.2291	2.7365	LPE 40:2	− 1.1076	4.7663
TAG 55:3|TAG 18:0_18:1_19:2	1.185	5.8993	TAG 46:6|TAG 10:0_14:0_22:6	− 1.1174	5.0592
TAG 47:2|TAG 14:0_14:0_19:2	1.1678	5.9837	SM 51:3;3O	− 1.1851	2.1625
TAG 8:0_8:0_15:0	1.161	7.6343	LPE 42:4	− 1.2039	5.6857
TAG 39:2|TAG 8:0_14:0_17:2	1.1299	7.8748	PE 8:0_23:0	− 1.37	2.3249
TAG 17:1_20:0_20:0	1.1241	5.0208	MAG 3:0	− 1.428	2.6604
Cer 30:3;2O/18:2	1.117	6.0743	PS 26:0_26:1	− 1.493	1.7246

**Figure 4:**
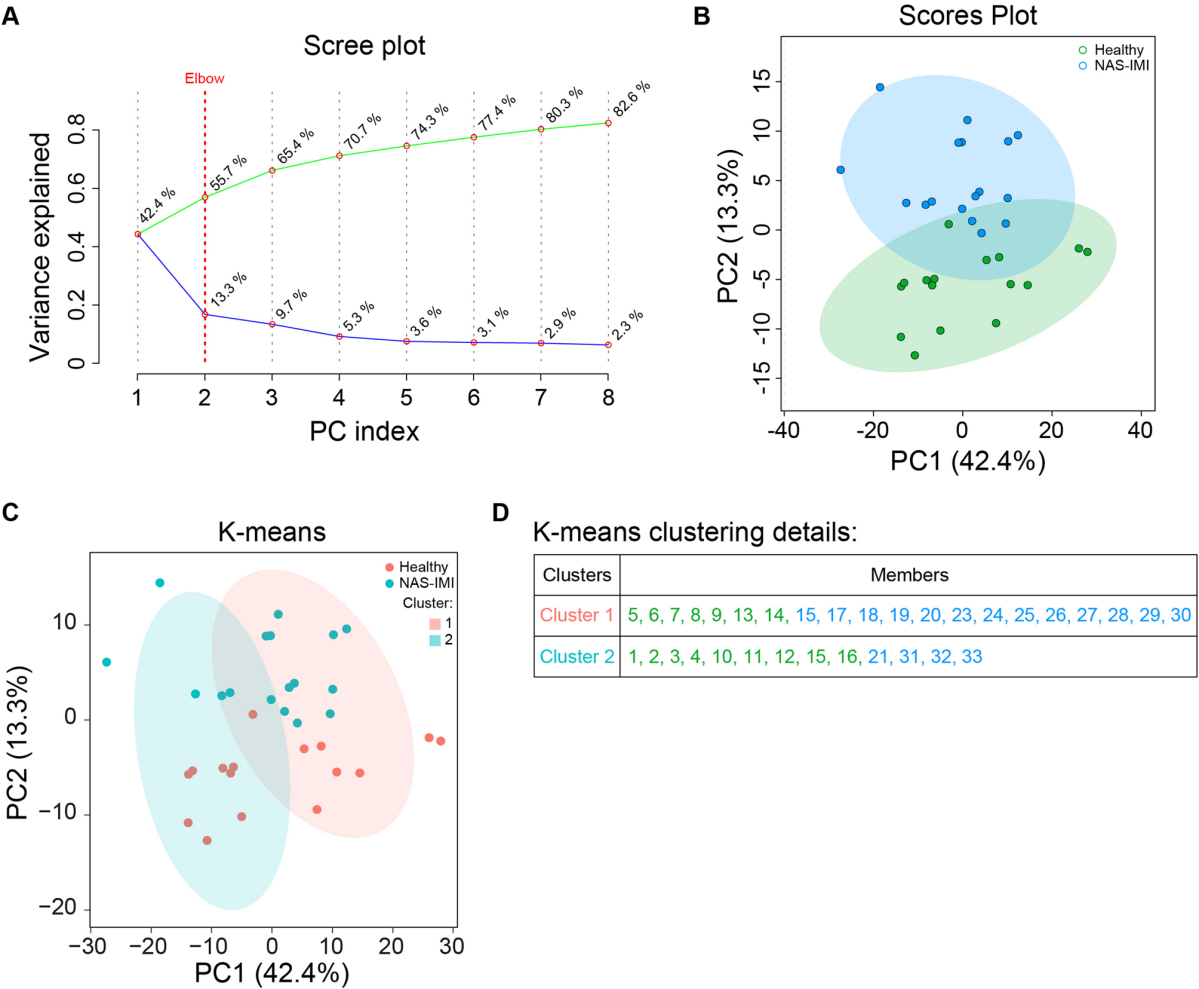
Lipidome discriminates between H and NAS-IMI milk. (**A**) Scree plot of the principal component analysis (PCA) representing the total variance (green line) and the single variance (blue line) explained for each component (PC index). (**B**) 2D plot of the PCA representing sample distribution based on PC1 and PC2 of lipidomic profiles of healthy (pink) and NAS-IMI (cyan) milk samples. (**C**) 2D plot of K-means clustering based on PC1 and PC1 from PCA. (**D**) Details of samples distribution among K-means clusters.

Figure 4 presents the Principal Component Analysis (PCA) analysis that measured H and NAS-IMI milk differences. The PCA scree plot demonstrated that PC1 and 2 explained 42.4% and 13.3% of the total dataset variance (Fig. 4A). Moreover, the results showed that scatters of the two groups were not separated across PC1 and PC2 (Fig. 4B). K-means clustering was performed to assess whether PC1 and PC2 could identify experimental groups; our results indicate that PC1 and PC2 cannot discriminate between the two populations (Fig. 4C, D).

**Figure 5:**
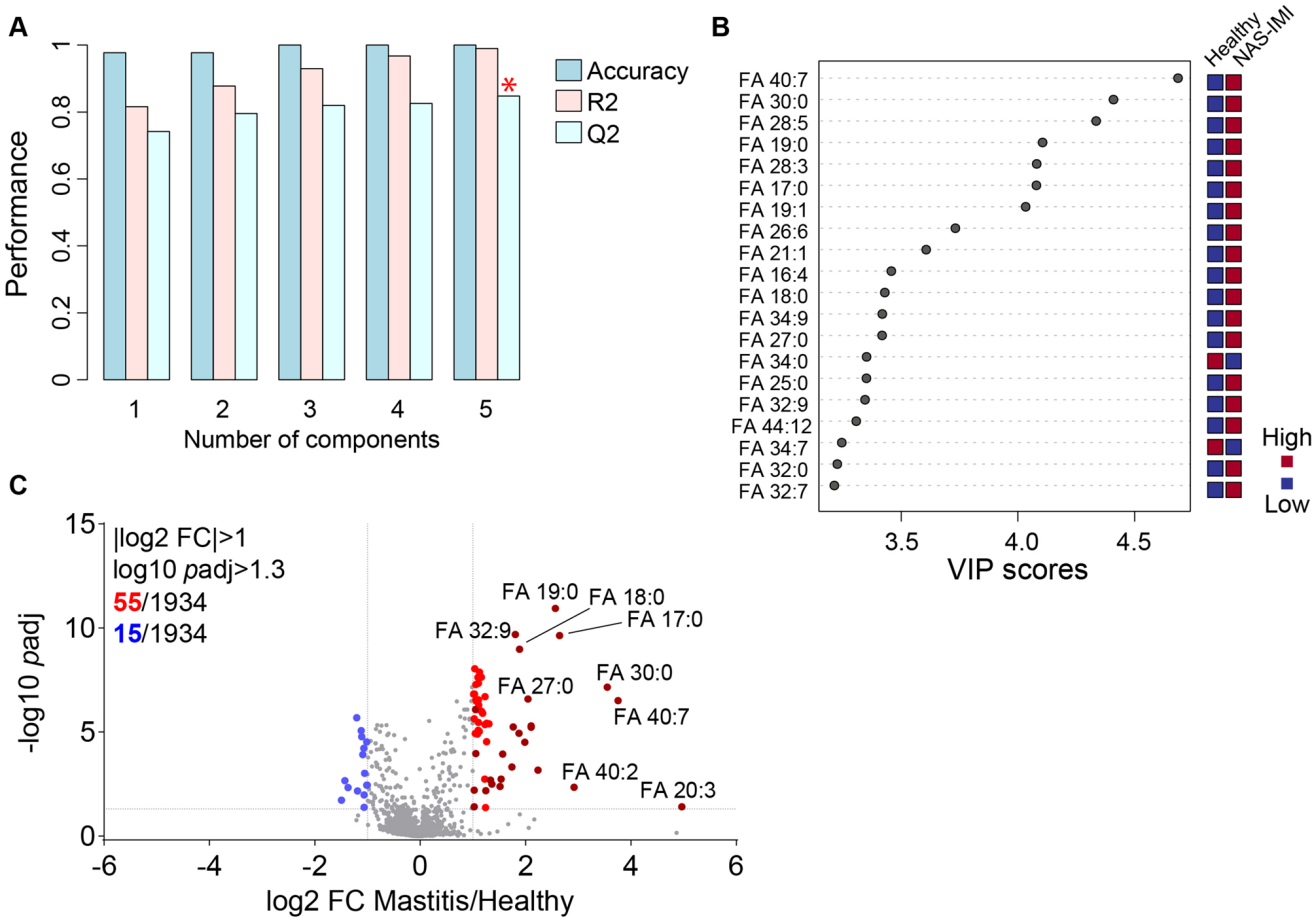
NAS-IMI impairs specific lipids in the milk of water buffalos. (**A**) Cross-validation performed by the tenfold method to estimate the predictive ability of the generated models. Asterisk on model no. 5 indicates the highest and most consistent model (highest Q2, where Q2 estimates the model's predictive ability). (**B**) Variable importance in projection (VIP) scores of indicated metabolites listed in model no. 5. Blue and carmine red squares indicate low and high lipid levels. (**C**) Volcano plot representing down—(blue dots), upregulated (red dots), and unchanged lipids (grey dots) in NAS-IMI compared to H milk. Dark blue and carmine red dots indicate differentially abundant fatty acids. Significant lipids were considered with |log2 fold-change|> 1 and − log10 adjusted *p* value > 1.3.

To gain further insights into potential differences between lipidome of H and NAS-IMI milk, we took advantage of Partial Least Square-Discriminant Analysis (PLS-DA). We found 2 out of 5 predictive models with a Q2 higher than 0.8, with component 5 showing the highest Q2 (Fig. 5A). The prediction model 5 indicated that fatty acids were the most important variables in our dataset; in fact, the 20 most important lipids were all fatty acids according to variable importance in projection (VIP) score, with the first 14 showing a VIP score > 3.4 (Fig. 5B). Bivariate analysis was constructed to confirm these findings using specific thresholds, namely |log2 fold-change|> 1 and − log10 adjusted *p* value > 1.3. The volcano plot identified 72 different lipid species in the milk of NAS-IMI compared to H animals, with 55 increased and 17 reduced lipids, respectively, in IMI compared to H quarters (Fig. 5C and Table 2). Most of the differentially abundant lipids were TAG and FA, which mostly increased in NAS-IMI compared to healthy milk samples (Fig. 5C and Table 2). Nevertheless, the concentration of some ceramides (Cer 8:1;2O/42:7, Cer 18:1;2O/44:8, Cer 26:0;2O/18:5, Cer 12:2;2O/22:1, Cer 30:3;2O/18:2), diacylglycerols (DAG 36:0 (8:0–28:0), DAG 63:8 (19:0–44:8)), sphingomyelins (SM 29:0;2O/2:0, SM 40:4;3O, SM 51:2;3O, SM 15:3;2O/42:8, SM 38:2;3O, SM 51:3;3O) and lysophosphatidylethanolamines (LPE 44:3, LPE 40:2, LPE 42:4) were also affected by IMI-NAS (Table 2).

**Figure 6:**
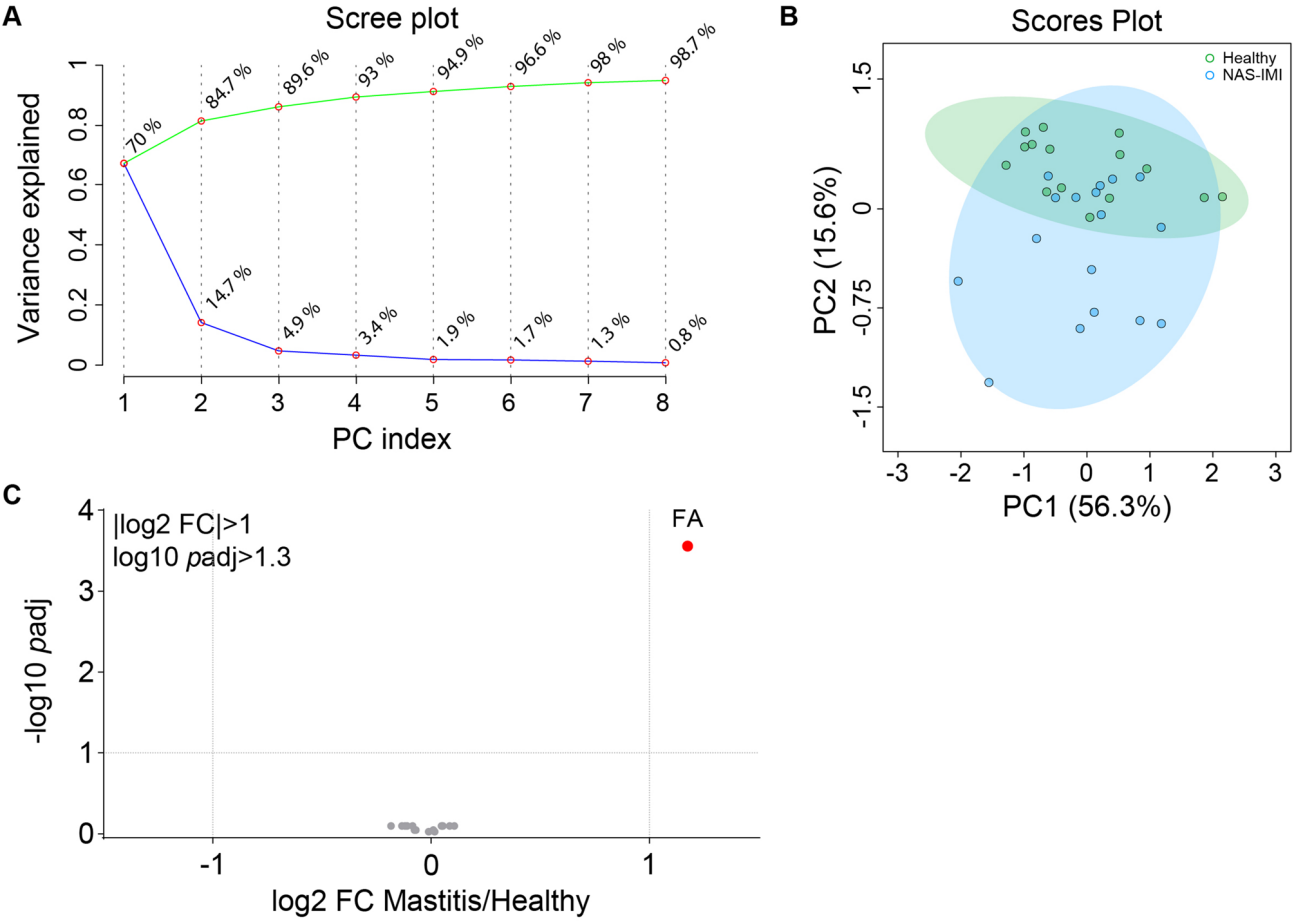
Lipid classes do not discriminate the lipidome of NAS-IMI and H water buffalo milk. (**A**) Scree plot of the principal component analysis (PCA) representing the total variance (green line) and the single variance (blue line) explained for each component (PC index). (**B**) 3D scatter plot of the PCA representing sample distribution based on PC1, PC2, and PC3 of lipidomic profiles of healthy (pink) and NAS-IMI (cyan) milk samples. (**C**) Volcano plot representing down-, upregulated (red dot) and unchanged lipid classes (grey dots) in NAS-IMI compared to H buffalos. Significant lipid classes were considered with |log2 fold-change|> 1 and − log10 adjusted *p* value > 1.3.

Finally, according to the main lipid classes, we investigated the difference between H and NAS-IMI milk. Following these criteria, the PCA analysis indicated that H and NAS-IMI quarter milk samples were similar in lipid classes (Fig. 6A, B). Consistent with the results in Fig. 5 and Table 2, the volcano plot identified FA as the only affected class in IMI compared to H milk (Fig. 6C).

## Discussion

In water buffalo, coagulase-negative staphylococci are considered one of the most frequent IMI agents. In the absence of information on how NAS-IMI alters the lipid profile of milk, the present study fills this gap by presenting the lipidome of water buffalo milk analyzed by untargeted liquid chromatography-quadrupole time-of-flight mass spectrometry (LC-QTOF-MS) and comparing healthy with IMI affected quarter milk. This is also the first study to investigate the untargeted lipidome content of healthy water buffalo milk, to the authors' knowledge. Previous studies focused on the metabolome of milk as an early predictor of pregnancy using the liquid chromatography-mass spectrometry analysis (LC–MS) approach^26^. Other studies examined the effects of nutrition on milk fatty acid profiles using high-resolution gas chromatography (GC) and identified 32 fatty acids^29,30^. Two other GC studies also identified 32 and 50 fatty acids, respectively, suggesting a similar number of lipid species^31,32^. The metabolome of water buffalo milk was recently studied using an OMICS approach (LC–MS/MS). However, the lipid subset identified in this study represented only a small portion of the total lipids^33^.

Of the 1854 lipid species reported in the present study, 72 were differentially abundant in milk from NAS-IMI quarters compared with healthy quarters, the fatty acids being the most affected lipid class.

In the first part of our study, we determined the lipidome of healthy milk. As expected, and as described for other species^34,35^, milk lipids are mainly composed of TAGs, with TAG 36:0, TAG 38:1, and TAG 34:0 being the major TAG species. Considering that this is the first study performed on water buffalo, comparisons with existing data cannot be made. After comparing with the closest comparable data set, the bovine milk lipidome^36^, we found that the most abundant bovine milk TAG are also present in water buffalo milk. The two primary fatty acids isolated from water buffalo milk are C16:0 (palmitic acid) and C18:0 (stearic acid), as described^26,32,33^. Remarkably, the presence of very long-chain fatty acids, namely FA 42:5 and FA 40:5, in milk, is reported here for the first time. On the contrary, the amount of C18:1 (oleic acid), previously identified as one of the main FA in water buffalo milk^26,32,33^, was not as abundant as others. Fatty acid 14:0 (myristic acid) was also not as abundant as previously reported^29,32^. The differences between the present results and those of other studies can be explained in part by the different techniques used to extract the lipids and the different feeding of the animals, given that the lipid content of the milk depends strongly on the cattle's diet^37^. In this study, healthy milk quarters with low (< 1 × 10^5^ cells/mL) and high SCC levels (> 1 × 10^5^ cells/mL) were collected, but PCA analysis did not reveal statistically significant changes associated with SCC in milk lipidome. According to this result, somatic cells have very limited influence on milk lipidome, confirming previous studies on bovine milk^36^.

Interestingly, we found no acylcarnitine molecule in water buffalo milk. This result contradicts other results where acylcarnitines were detected in Mediterranean water buffalo milk^33^, although the higher concentration was found in milk samples from non-pregnant water buffaloes and in a lesser amount in pregnant cows. It can be hypothesized that acylcarnitine levels during mid-lactation were below the detection limit of the technique and the DIM condition of the water buffaloes involved in the study. However, in a recent study, acylcarnitine was detected in the milk of buffaloes during parity and lactation, similar to the present study^33^. It can also be ruled out that the method used for lipid extraction from water buffalo milk did not extract acylcarnitine molecules, as 25 acylcarnitine molecules were identified in our study of the lipidome of bovine milk using the same method^36^.

Compared to cow milk, the number of complex lipids, such as TAG and DAG, is relatively low (70% and 64% for TAG and DAG, respectively). A decrease was observed for SM and Cer (80% and 30%, respectively), while PG, PE, and AcCarn were not detected. Buffalo milk is particularly rich in fatty acids (94% more than cow milk) and HexCer, a lipid class not found in cow milk, while water buffalo milk contains 145 different species. There are differences in the ratio of Cer to HexCer between cow's milk and water buffalo milk. In cow's milk, the ratio is 348:0, while in water buffalo milk, the ratio is 148:164. In addition, Ceramide 1 Phosphate is present in buffalo milk but not in cow milk. CerP is a ceramide metabolite formed by ceramide kinase after direct phosphorylation of ceramide^38^. CerP has a profound effect on the physiology and functions of immune defence cells. CerP regulates proliferation and apoptosis of bone macrophages^38^ by upregulating anti-apoptotic proteins such as BcL-X_L_^39^. Remarkably, the nonphosphorylated form of ceramide has an opposite downregulatory effect on BcL-X_L_^40^ and increases alveolar macrophage apoptosis^41,42^. In addition, CerP stimulates the release of arachidonic acid and the synthesis of prostanoids and proinflammatory cytokines, as demonstrated in an adenocarcinoma cell model^43^, and enhances immune defence by stimulating neutrophil phagocytosis^44^, mast cell degranulation^45^, and macrophage chemotaxis^46^. Finally, CerP can prevent LPS-induced injury by attenuating NFK-B activity^47^. Because CerP is involved in regulating the immune system, it can be assumed that CerP differences are one of the factors that could explain the different responses of water buffalo to NAS-IMI compared to dairy cows.

The second step of the study was to determine the lipidome composition of NAS-IMI milk compared to the lipidome of H milk. The NAS-IMI changed the abundance of 72 lipid species, of which 65 species increased and 17 species decreased, with most of them being free fatty acids class. It has been previously reported that fatty acids are increased during mastitis^48^ due to the susceptibility of milk fat to lipase activity^49^ and lipolytic activity of *S. aureus*, which leads to increased fatty acids levels^50^. The PCA analysis cannot distinguish between H and NAS-IMI quarters based on lipid classes. These results contrast with our previous studies on bovine milk containing NAS-IMI, in which groups of quarter milk containing H- and NAS-IMI could be separated^36^. In similar studies carried out in dairy cows, we found that NAS-IMI induced an increase of 597 lipids compared with healthy quarters. The changes that we identified in the present study on water buffalo are sensibly different from bovine milk, being limited to 72 lipids species that are differentially abundant in the NAS-IMI affected milk. Although water buffaloes are regarded as less subjected to mastitis, this concept is debatable, as mastitis is the most prevalent disease in this species, in both farming conditions where animals are kept in an intensive dairy milk productive system^22^, or where the hygienic environment is suboptimal^51^. Still, at least for what concerns the specific NAS-IMI and the lipidome content, the water buffalo seems much less sensitive to dairy cows, given that our results showed that NAS-IMI induced limited changes in the lipid profile of affected quarter milk. Further studies are indeed required, at both clinical and molecular levels, to understand the real impact of NAS-IMI in weater buffaloes.

The authors acknowledge that the experimental design has some limitations. Only a small number of samples were examined in this study based on an untargeted approach. Therefore, the results should be validated by a quantitative targeted approach on a more significant number of milk samples. Animals recruited for the study were homogeneous concerning DIM (mid-lactation, 4th to 5th month) but not parity. While no primiparous animals appeared in the study, parity was detected from the second to the seventh lactation, with three samples, H13, H14, and H15, belonging to animals in their eleventh lactation. Finally, samples were obtained from quarter milk. There is no information on the independence of each quarter from adjacent quarters in water buffalo, except for the microbiome^17^. In dairy cows, each quarter is anatomically independent and has its vascular system, nervous system, and suspensory apparatus, suggesting that they may be considered independent^52,53^, suggesting that they can be regarded as independent. On the other hand, there is increasing evidence that infection in one udder quarter also affects healthy quarters^54^ and that the immune response may develop across quarters^55^. However, several studies still consider individual quarters as independent entities^56,57^.

## Conclusions

This was the first study to perform lipidome analyses on water buffalo milk in H and NAS IMI quarters using a LC-QTOF-MA approach. We identified 1854 lipids belonging to 14 classes and discovered the changes of 72 lipids in NAS-IMI compared to H milk, mainly belonging to the long-chain fatty acids class. Compared to bovine milk, the lipidome of water buffalo milk contains ceramide metabolites such as CerP, which play an important role in modulating innate immunity. The changes in the water buffalo lipidome during NAS-IMI are also minimal compared to the changes in bovine milk during NAS-IMI. Further studies are needed to validate and quantify the findings obtained in this study in a larger milk sample and confirm the effects of long-chain fatty acids and CerP on water buffalo immune defences.

## Methods

### Ethical statement

Samples were collected during the routine collection of milk samples for microbiological analysis related to mastitis control and milk quality. The samples included in the present study were collected from Italian Mediterranean Water Buffaloes (*Bubalus bubalis*) within the frame of a diagnostic routine collection of samples for microbiological analysis for monitoring the herd's health status. The lipidome was determined on 1 mL of leftover milk. This practice is approved by the Ethical Committee of the University of Milan (Comitato Etico 15.02.16 Parere numero 2/16), “allowing the use, under informed consent of the owners, of the residual volume of samples for studies on metabolic biomarkers”. All methods and procedures were performed following the relevant institutional guidelines and regulations. The methods described in the current study and reported results were compliant with ARRIVE guidelines for reporting animal research^58^.

### Animal selection and collection of milk samples

The study was conducted on a commercial water buffalo dairy farm in Campania Region, South Italy. The farm housed an average of 270 milking water buffaloes housed in free stalls in deep-bedded cubicles with straw. All animals were fed a balanced Total Mixed Ration in feed alleys with headlocks. Lactating cows were milked twice daily in a double 12 herringbone milking parlour. Milk samples were collected according to the guidelines of the National Mastitis Council^59^. Before sampling, all teat ends were carefully cleaned with a pre-dipping foam containing lactic acid, and the apex was disinfected with alcohol. First, the foremilk streams were discharged, and then 20 mL of milk from each teat was aseptically poured into sterile vials. Samples were stored at 4 °C until bacteriological tests and somatic cell count (SCC) were performed.

### Sample selection

The study was conducted on quarter milk samples. The samples included in the experimental design are listed in Supplementary Table S1 and were collected from multiparous cows in their mid-lactating period. The collected samples were classified as follows:

A1: healthy (H) quarters (13 samples). These milk samples had SCC < 100.000 cell/ml and were negative for udder pathogen growth.

A2. Quarters with high somatic cells (> 100,000) (HSCC) but no clinical signs and negative microbiological analysis (3 samples).

B: Intramammary Infected (IMI) quarters (17 samples), with SCC > 100 × 10^3^/ml, using a threshold of at least five NAS colonies isolated from a 10-μL milk sample^60^.

### Somatic cells count and microbiological culture of milk

Somatic cell count (SCC) was measured in milk samples using the Fossomatic (Foss) apparatus using the UNI EN ISO 13366-2: 2007 technique for electronic, optical fluorometric counters. Bacteriological cultures were performed according to the National Mastitis Council guidelines^59^ as previously reported, ^15^ on 10 ml each of milk. Briefly, cultures were incubated for 24 h at 37 °C under aerobic conditions on blood agar (Trypticase Soy Agar containing 5% defibrinated sheep blood), MacConkey agar, and Baird Parker Agar; for 72 h at 37 °C under aerobic conditions on Prototheca Isolation Medium (PIM) and under micro-aerobic conditions on Mycoplasma agar. Gram stain, coagulase, and oxidase assays were performed on positive cultures, particularly *Staphylococcus* spp. Detection of coagulase in cultures was performed with rabbit plasma and then for *Streptococcus* spp. The Streptokit-BioMérieux assay was applied using Lancefield grouping to identify antigen differences between species^15^.

### Preparation of milk samples for lipidomic analysis: lipid extraction

The preparation of samples was carried out as previously described according to the Folch method^61^, with few changes^36^. Briefly, each sample's two aliquots (100 µL) were added with internal standards and extracted. Internal standards were 13-Docosenamide for positive ion mode, 13C-palmitic acid and 13C-linoleic acid for negative ion mode^62^. The organic residue was reconstituted with 200 µL of 2-propanol: acetonitrile (90:10, v/v), 0.1% formic acid, and 10 mM ammonium acetate. Aliquots of 20 µL were then diluted 1:10 with mobile phase B for lipidomic analysis in positive mode. Aliquots of 5 µL were analyzed in negative ion mode for free fatty acid analysis.

### Lipidomic analysis

Lipidomic analysis was carried out as already described in^36^. Briefly, 2 and 5 µL of the sample were separated for positive and negative ion modes, respectively, by liquid chromatography (LC) using a Kinetex EVO C18—2.1 × 100 mm, 1.7 µm (Phenomenex®) column at 45 °C connected to an ExionLC™ AD system (ABSciex) maintained at 15 °C. The separated lipids were then ionized using an electrospray ionization (ESI) source and analyzed in a TripleTOF 6600 (Quadrupole Time-Of-Flight, QTOF—ABSciex) mass spectrometer. The mobile phases were A: water with 0.1% formic acid and 10 mM ammonium acetate/acetonitrile (60:40); B: 2-propanol with 0.1% formic acid and 10 mM ammonium acetate/acetonitrile (90:10). The following elution gradient was used: 0 min, 55% B; 2 min 55% B; 12 min, 3% B; 17 min, 3% B; 17.10 min, 55% B; 20 min, 55% B. The flow rate was 0.4 mL/min. The ESI and mass spectrometer conditions were set as shown in Supplementary Table S6.

### Data processing

Data were expressed as the ratio of the analyte to the internal reference (1-Phenoxy-2-propanol). We processed the data with untargeted data processing software MSDIAL (v3.98), using the LipidBlast database v2019. The database contains 143342 MS/MS spectra of 110833 analytes divided into 32 lipid classes. A cut-off score of 80% was used for lipid identity match. After processing, we identified a total of 1934 lipids species. It must be pointed out that the automated annotation of metabolites and lipids is a significant issue and the identification via these tools remains tentative. Lipids were classified into 15 classes, including free fatty acids (FA), monoacylglycerols (MAG), diacylglycerols (DAG), triacylglycerols (TAG), phosphatidylserine (PS), phosphatidylinositol (PI), sphingomyelins (SM), Phosphatidylcholine (PC), lysophosphatidylcholines (LPC), hexosyl ceramides (HexCer), lysophosphatidylethanolamine (LPE), phosphatidylethanolamine (PE), ceramide phosphate (CerP), ceramide (Cer), cholesterol esters (CE).

### Statistical analysis

MetabolAnalyst 4.0 Webtool was used to analyze the data. Variables containing more than 20% of missing values (i.e. values less than LOD) were not considered for the statistical analysis^63^. Missing values were imputed with Bayesian Principal Component Analysis (BPCA). Afterwards, the data were transformed using a generalized log-transformation, and Pareto scaled to account for heteroscedasticity, reduce the data's skewness, and eliminate mask effects^64^. The Principal Component Analysis (PCA), the Partial Least-Squares Discriminant Analysis (PLS-DA) and the volcano plots and heatmaps were generated using MetaboAnalyst 5.0 web tool. PLS-DA is a supervised method that can perform both classification and feature selection and was used to identify the most representative lipid species in NAS-IMI milk samples compared to controls. Volcano plots were created to provide an overview of the significantly affected lipids. Volcano plots display the size of the biological effect (fold-change) versus the statistical significance of the result. Important lipids were identified by the volcano plot based on the fold change threshold of 2.0 on the x-axis and the t-tests threshold [false discovery rate (FDR) adjusted *p* value] of 0.05 on the y-axis.

## Supplementary Information

Supplementary Figures.

Supplementary Legends.

Supplementary Table S1.

Supplementary Table S2.

Supplementary Table S3.

Supplementary Table S4.

Supplementary Table S5.

Supplementary Table S6.

## Data Availability

All data generated or analysed during this study are included in this published article [and its supplementary information files].
