# Supplementary figures and images for: Changes in the lipidome of water buffalo milk during intramammary infection by non-aureus Staphylococci
Source: Sci Rep. 2022 Jun 11;12:9665. doi: 10.1038/s41598-022-13400-0 (PMC9188581; doi:10.1038/s41598-022-13400-0)

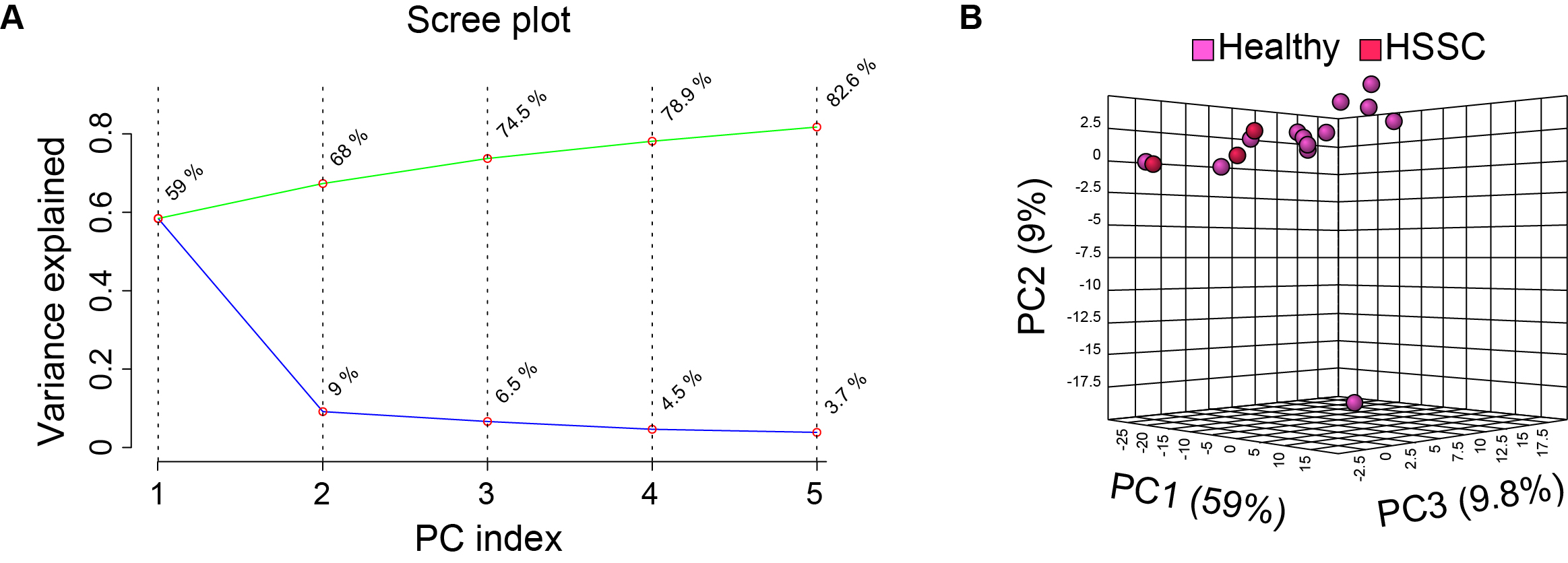

Supplement: Supplementary file 1 — Supplementary Figures. [file 41598_2022_13400_MOESM1_ESM.jpg]
