# Supplementary material for: Changes in the lipidome of water buffalo milk during intramammary infection by non-aureus Staphylococci
Source: Sci Rep. 2022 Jun 11;12:9665. doi: 10.1038/s41598-022-13400-0 (PMC9188581; doi:10.1038/s41598-022-13400-0)
Supplement: Supplementary file 2 — Supplementary Legends. [file 41598_2022_13400_MOESM2_ESM.docx]

Supplementary Figure 1. Lipidomic profile of high somatic cell count (HSSC) quarters (SCC >100000) is unaffected compared to healthy (SCC <100000) samples. A) Scree plot of explained variance and B) 3D-PCA of healthy (pink dots) and HSCC (red dots) samples.
