# Supplementary material for: Changes in the lipidome of water buffalo milk during intramammary infection by non-aureus Staphylococci
Source: Sci Rep. 2022 Jun 11;12:9665. doi: 10.1038/s41598-022-13400-0 (PMC9188581; doi:10.1038/s41598-022-13400-0)
Supplement: Supplementary file 3 — Supplementary Table S1. [file 41598_2022_13400_MOESM3_ESM.docx]

**Supplementary Table S1**. Schematic description of the samples used in lipidomic analysis

| **Animal** | **Quarter** | **Sample identification** | **SCC** | **Clinical Status** | **Parity** | **DIM** |
| --- | --- | --- | --- | --- | --- | --- |
| 719 | FL | H1 | 51000 | Healthy | 6 | 144 |
| 818 | FL | H2 | 99000 | Healthy | 5 | 95 |
| 787 | RL | H3 | 73000 | Healthy | 5 | 113 |
| 746 | FR | H4 | 36000 | Healthy | 5 | 120 |
| 746 | RL | H5 | 48000 | Healthy | 5 | 120 |
| 884 | RR | H6 | 54000 | Healthy | 4 | 122 |
| 479 | FL | HSSC7 | 220000 | Healthy HSCC | 7 | 141 |
| 479 | RL | HSSC8 | 150000 | Healthy HSSC | 7 | 141 |
| 41 | FR | H9 | 30000 | Healthy | 2 | 90 |
| 41 | RR | H10 | 21000 | Healthy | 2 | 90 |
| 41 | FL | H11 | 20000 | Healthy | 2 | 90 |
| 993 | FL | H12 | 27000 | Healthy | 3 | 87 |
| 93 | RR | H13 | 24000 | Healthy | 11 | 91 |
| 93 | FL | H14 | 34000 | Healthy | 11 | 91 |
| 93 | RL | H15 | 75000 | Healthy | 11 | 91 |
| 59 | FR | HSSC16 | 290000 | Healthy HSSC | 2 | 103 |
| 719 | RL | IMI1 | 246000 | IMI | 6 | 144 |
| 818 | FR | IMI2 | 151000 | IMI | 5 | 95 |
| 787 | FR | IMI3 | 156000 | IMI | 5 | 113 |
| 787 | FL | IMI4 | 211000 | IMI | 5 | 113 |
| 787 | RR | IMI5 | 233000 | IMI | 5 | 113 |
| 65 | FR | IMI6 | 290000 | IMI | 2 | 114 |
| 65 | FL | IMI7 | 247000 | IMI | 2 | 114 |
| 754 | FR | IMI8 | 367000 | IMI | 6 | 128 |
| 754 | FL | IMI9 | 248000 | IMI | 6 | 128 |
| 754 | RR | IMI10 | 242000 | IMI | 6 | 128 |
| 754 | RL | IMI11 | 210000 | IMI | 6 | 128 |
| 884 | RL | IMI12 | 335000 | IMI | 4 | 122 |
| 61 | FR | IMI13 | 147000 | IMI | 2 | 132 |
| 829 | FR | IMI14 | 457000 | IMI | 5 | 204 |
| 829 | RR | IMI15 | 190000 | IMI | 5 | 204 |
| 829 | RL | IMI16 | 413000 | IMI | 5 | 204 |
| 993 | RR | IMI17 | 500000 | IMI | 3 | 87 |

RR - Rear- Right, RL – Rear Left, FR – Front Right, FL – Front Left; 1: number of somatic cells in 1 ml

DIM - Day in Milking
