# Supplementary material for: Changes in the lipidome of water buffalo milk during intramammary infection by non-aureus Staphylococci
Source: Sci Rep. 2022 Jun 11;12:9665. doi: 10.1038/s41598-022-13400-0 (PMC9188581; doi:10.1038/s41598-022-13400-0)
Supplement: Supplementary file 8 — Supplementary Table S6. [file 41598_2022_13400_MOESM8_ESM.docx]

**Supplemental Table S6:** ESI and mass spectrometer parameters.

| Item | Lipidomic IDA POS | Lipidomic IDA NEG |
| --- | --- | --- |
| Ionization | POS | NEG |
| Source temperature | 350 °C | 350 °C |
| Curtain Gas (CUR) | 35 | 35 |
| GS 1 | 55 | 55 |
| GS 2 | 65 | 65 |
| Ion Spray Voltage | 5500 V | -4500 V |
| Declustering Potential (DP) | 50 V | -50 V |
| Collision Energy | 35V | -40V |
| Collision Energy Spread | 15 | 20 |
| TOF MS Mass Range | 140-2000 Da | 150-1100 Da |
| IDA acquisition Mass Range | 50-2000 Da | 50-2000 Da |
| Top N | 18 | 10 |
